# D-stroi – a uniaxial load frame for X-ray diffraction and imaging

**DOI:** 10.1107/S1600577526005266

**Published:** 2026-06-18

**Authors:** Felix Tristan Frankus, Adam André William Cretton, Carsten Detlefs, Flemming Bjerg Grumsen, Antonella Gayoso Padula, Grethe Winther

**Affiliations:** a Department of Civil and Mechanical Engineering Technical University of Denmark Denmark; b Department of Physics Technical University of Denmark Denmark; c European Synchrotron Radiation Facility France; NSRRC, Taiwan

**Keywords:** plastic deformation, mechanical testing, dark-field X-ray microscopy

## Abstract

We present a remotely controlled miniature load frame for *in situ* tensile deformation of millimetre-sized metal samples in synchrotron X-ray diffraction and imaging environments. The load frame features in-built digital image correlation-based controls to accurately track deformation until rupture at tensile loads up to 500 N. The mechanical setup is designed to minimize the shift in the sample’s centre during loading, thereby maintaining a constant illumination volume.

## Abstract 2

A remote miniature load frame for X-ray diffraction and imaging applications is presented. The load frame has been assessed for mechanical stability and suitability for various synchrotron X-ray-based techniques.

## 1. Introduction

Tensile testing is a fundamental method for assessing the mechanical properties of materials (Gdoutos & Konsta-Gdoutos, 2024 ▸). Many material parameters important for describing the mechanical characteristics, such as yield point and ultimate tensile strength, are extracted from stress–strain curves obtained from such tests (Davis, 2004 ▸). As the sample is tensile-deformed, its microstructure changes to accommodate the imposed shape change. These changes can be characterized using synchrotron-based X-ray diffraction techniques at multiple length scales. Dark-field X-ray microscopy (DFXM) is a diffraction-contrast-based imaging technique used to probe intragranular orientation distributions within crystalline materials (Simons *et al.*, 2015 ▸). Tracking their evolution during tensile deformation requires an appropriate *in situ* tensile stage that is compatible with the sample goniometer and the beamline environment.

A manually operated miniature load frame was used by Lee *et al.* (2025 ▸) in an *in situ* DFXM study on twin formation in magnesium. This setup uses dovetail-shaped removable sample grips, which serve as the mounting interface. Other dedicated setups for synchrotron environments include miniature setups such as the motor-controlled Nanox (Gueninchault *et al.*, 2016 ▸), which employs an X-ray-transparent glass tube to support the reaction force and provide the rotational degrees of freedom necessary for three-dimensional X-ray diffraction (3DXRD) [high-energy diffraction microscopy (HEDM)] and diffraction contrast tomography (DCT), and is not yet coupled to measurement of the sample elongation by, for example, digital image correlation (DIC). A different design approach is followed by the rotational and axial motion system (RAMS) which serves as an insert for conventional mechanical load frames. The insert enables rotation of the sample around its tensile axis under applied loads (Shade *et al.*, 2015 ▸). RAMS is designed for 3DXRD (HEDM) and has many advantages in terms of stability, but it is also too bulky and heavy for some synchrotron setups.

Experiments employing small beam cross sections, as in DFXM where a focused line beam of ∼500 nm height is typically used, place stringent requirements on the mechanical stability of the setup. Maintaining this stability during tensile deformation is challenging as elongation inevitably induces sample motion. This elongation-induced systematic drift can be minimized by a symmetric loading configuration. By applying equal displacements to both sample ends, the central region remains approximately fixed.

**Figure 1:**
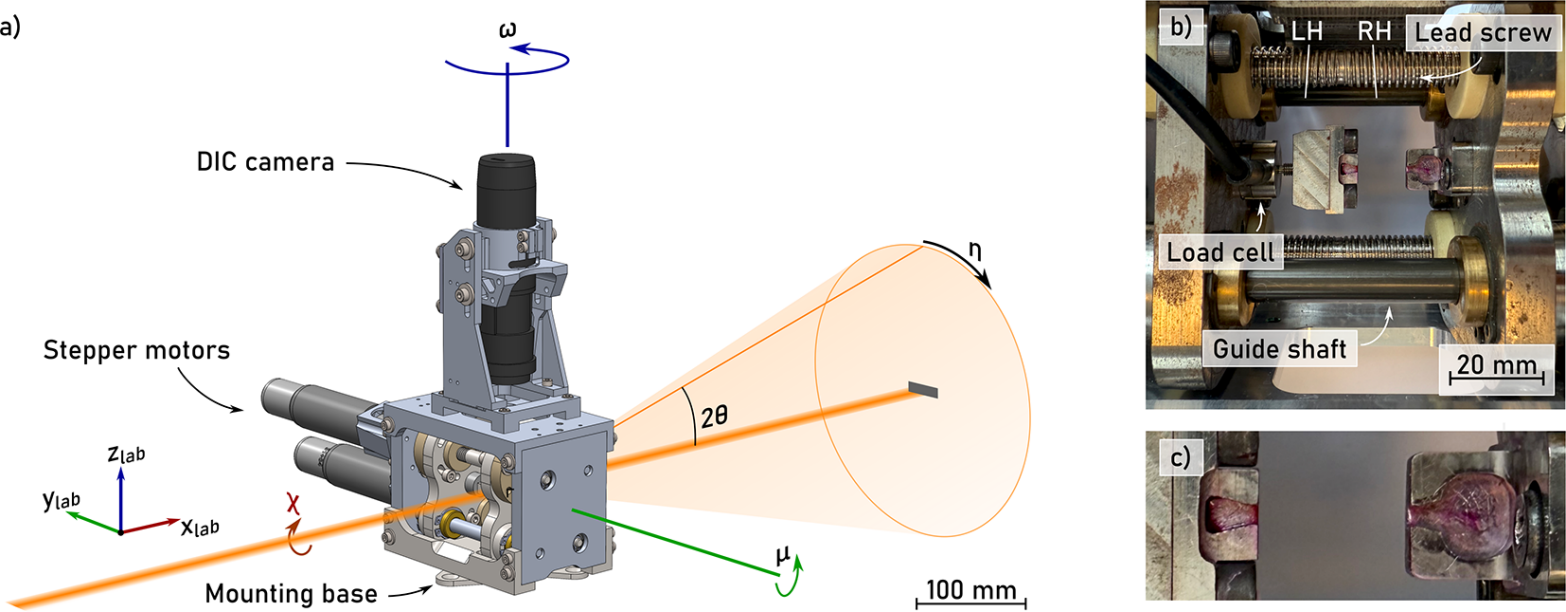
(*a*) Rendering of the load frame and diffraction geometry. The incident X-ray beam is parallel to *x*_lab_. The diffraction condition of the sample is changed by rotation of the entire load frame (sample) around axes *y*_lab_ and *z*_lab_ by angles μ (left-handed rotation convention) and ω, respectively. (*b*) Photograph of the sample environment taken along the beam direction. (*c*) Magnified view of the dovetail-shaped sample mounts.

The presented load frame – d-stroi – ensures symmetric loading by the coupled motion of both crossheads (*cf.* Fig. 1 ▸). Similar design approaches are available for miniature load frames for optical and scanning light electron microscopes (*cf.* the motor-actuated tensile and compression module by Kammrath & Weiss GmbH). Larger and heavier dual lead-screw stages for transmission geometries are also commercially available (*cf.* MT2000DL by Deben UK Ltd). However, it is challenging to find miniature devices with symmetric loading that combine high angular acceptance with compact dimensions and minimal weight.

To allow for monitoring and control of the straining process during synchrotron measurements, the presented load frame can be remotely operated from the control room via a network connection. The macroscopic elongation of the sample is recorded in real time using digital image correlation of images collected by a dedicated microscope camera. Elongation increments of down to Δɛ = 0.02% are possible, allowing for imaging effects arising from atomic-level defects in metals (dislocations) that are migrating in response to the mechanical loading. The load frame has been successfully employed in *in situ* DFXM experiments to track microstructural evolution in aluminium (Cretton *et al.*, 2025 ▸).

While the load frame is designed for the diffraction geometry and specifications of the DFXM setup at the ID03-HXM beamline at the European Synchrotron Radiation Facility (ESRF) (Isern *et al.*, 2025 ▸), its design is compatible with experiments at other sources, such as X-ray free-electron lasers and neutron facilities.

## 2. Load frame design

### 2.1. Mechanical design

The mechanical design of the load frame features two movable crossheads, with the sample mounted between them. Crosshead motion is produced by stepper-motor-driven lead screws. The spindles feature both right- and left-handed threads [*cf.* Fig. 1 ▸(*b*) and Fig. B.3 of the supporting information] by which each of the crossheads is driven through polymer nuts to assure coupled motion of the two. The maximum axial load of the nuts of 900 N is limiting the frame’s load capacity to 1800 N. The crossheads are guided by brass bearings running on two shared aluminium shafts. While the load-bearing components are machined from construction steel, the frame consists of aluminium components to ensure reaching the maximum weight of 1.8 kg. An 8 MP optical camera (Dino-Lite AM8117MZT) imaging the gauge area of the sample is mounted to the frame to monitor the sample’s elongation via DIC.

The step motor output is reduced through planetary gearboxes with a ratio 592:1 which, combined with the lead screw pitch of 1.5 mm, gives rise to a nominal minimum step size of 211 nm per motor step. At a stepping frequency of 400 Hz and a sample length of 10 mm, the strain rate amounts to 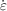 = 1.7 × 10^−2^ s^−1^. The reaction force is measured by a load cell between the upper crosshead and sample mount. The load cell in the current setup provides a maximum capacity of 500 N and is interchangeable with an identical model with a maximum load of 1 kN. A list of the mechanical components is given in Table A.1 of the supporting information.

### 2.2. Sample geometry

The load frame uses a customized sample geometry as shown in Fig. B.1 of the supporting information. The sample geometry features a parallel length *L*_C_ of 10 mm and a squared original cross-sectional area *S*_0_ of 1 mm^2^. By exchanging the sample mounts, larger sample cross-sections can be accommodated. The original gauge length *L*_0_ is 5.65 mm, following the proportional test piece definition of ISO 6892-1:2009. To prevent deformation of soft materials during handling and insertion, the sample is equipped with a 3.5 mm-long pin for gripping outside the gauge area. The mounting is ensured by dovetail-shaped ends with a slope of 1:2, eliminating the need for mechanical clamping. Avoiding plastic deformation during sample insertion and mounting is of high importance for experiments, for example, on the early stages of plastic deformation. After being inserted into the sample-mount pockets [*cf*. Fig. 1 ▸(*c*)] and preloaded, the specimen is further secured using cyanoacrylate. The sample geometry is established by wire-cut electrical discharge machining (EDM).

### 2.3. Digital image correlation

The DIC camera features a sensor with 3840 × 2160 pixel resolution and is equipped with active lighting and a polarization filter to suppress reflections from the sample surface. The camera is oriented such that the tensile axis is parallel to the longer side of the pixel array. The working distance is chosen such that the unstrained samples’ parallel length covers approximately 2000 pixels of the field of view. This allows for a maximum elongation of approximately 90% under the condition that the whole parallel length of the strained sample shall remain in the field of view. At this setting, the effective pixel size is 5 µm, resulting in a nominal strain resolution of 0.088% per pixel.

The used algorithm evaluates the elongation of the sample through tracking the relative displacement of two set patches (fiducials) on the sample’s surface. Each patch is a rectangular subset of the image with a size of 20 × 20 pixels. To increase the robustness of the image correlation, tests with random dot patterns were conducted; however, surface roughness from the EDM process has proven sufficient to provide the algorithm with enough features. Images generated by the setup are presented in Fig. B.2 of the supporting information. The image correlation is based on OpenCV’s implementation of the Lucas–Kanade optical flow algorithm (Bradski, 2000 ▸; Lucas & Kanade, 1981 ▸).

### 2.4. Periphery

**Figure 2:**
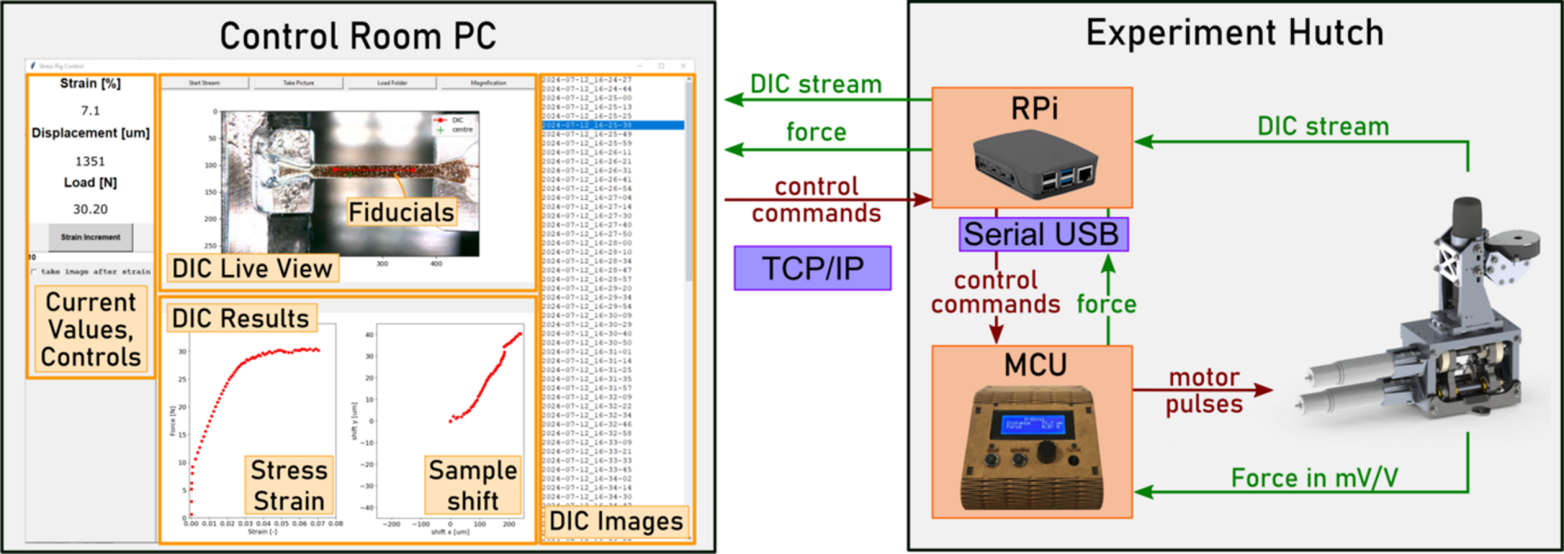
Scheme of the control components and data flow. Left: control software with graphical user interface. Right: setup in the experimental hutch comprising MCU, Raspberry Pi and load frame

A schematic of the control setup for the load frame is shown in Fig. 2 ▸. It features two microcontrollers [Motor Control Unit (MCU) and Raspberry Pi] placed along the load frame inside the experimental hutch, and a client software with a graphical user interface (GUI) running from the beamline’s control room. Communication between the client software and the microcontroller inside the hutch is via a network using TCP/IP.

The MCU supplies current to the step motors and reads the load cell. An Arduino Uno microcontroller serves as the MCU’s logic unit. The phase currents for the step motors are generated by a dedicated X-Nucleo-IHM02A1 step motor controller. Likewise, the load cell is operated by a dedicated microcontroller board (SparkFun Qwiic Scale) that generates the excitation voltage and converts the output voltages using a 24-bit A/D converter (NAU7802). To allow stand-alone control of the load frame, the unit features two push-buttons, a rotary encoder and a dot-matrix display (HD44780). Internal communication between the components is provided via an I^2^C bus. The housing is made of laser-cut medium-density fibreboard (MDF). The motors and the force sensor cables are connected to the MCU via four- and five-pin XLR connectors. A list of components is presented in Table A.2 of the supporting information.

TCP/IP communication with the control room software is established by a Raspberry Pi connected via USB to the Arduino inside the MCU. The USB port serves as an alternative input to the MCU’s push-buttons. Additionally, the microscope camera is connected to the Raspberry Pi. The Raspberry Pi continuously streams a live view of the camera and the current load cell output to the control room software and receives load instructions.

### 2.5. Remote control software

The load frame is controlled from the beamline control room via a GUI. A screenshot of the GUI is shown on the left of Fig. 2 ▸. It features a live view from the DIC camera, a printout of the current load and crosshead positions, and results of the DIC evaluation, including the current strain, a stress–strain curve, and a graph of the sample’s centre drift. The positions of the fiducials used for DIC are overlaid in the live-view and can be set there.

The user specifies the load step in a text field and initiates the increment with a button. After a load increment, a full-sized picture from the stream is saved, and the DIC program is executed. Once the DIC evaluation is complete, the strain values and plots are updated.

### 2.6. Angular acceptance

**Figure 3:**
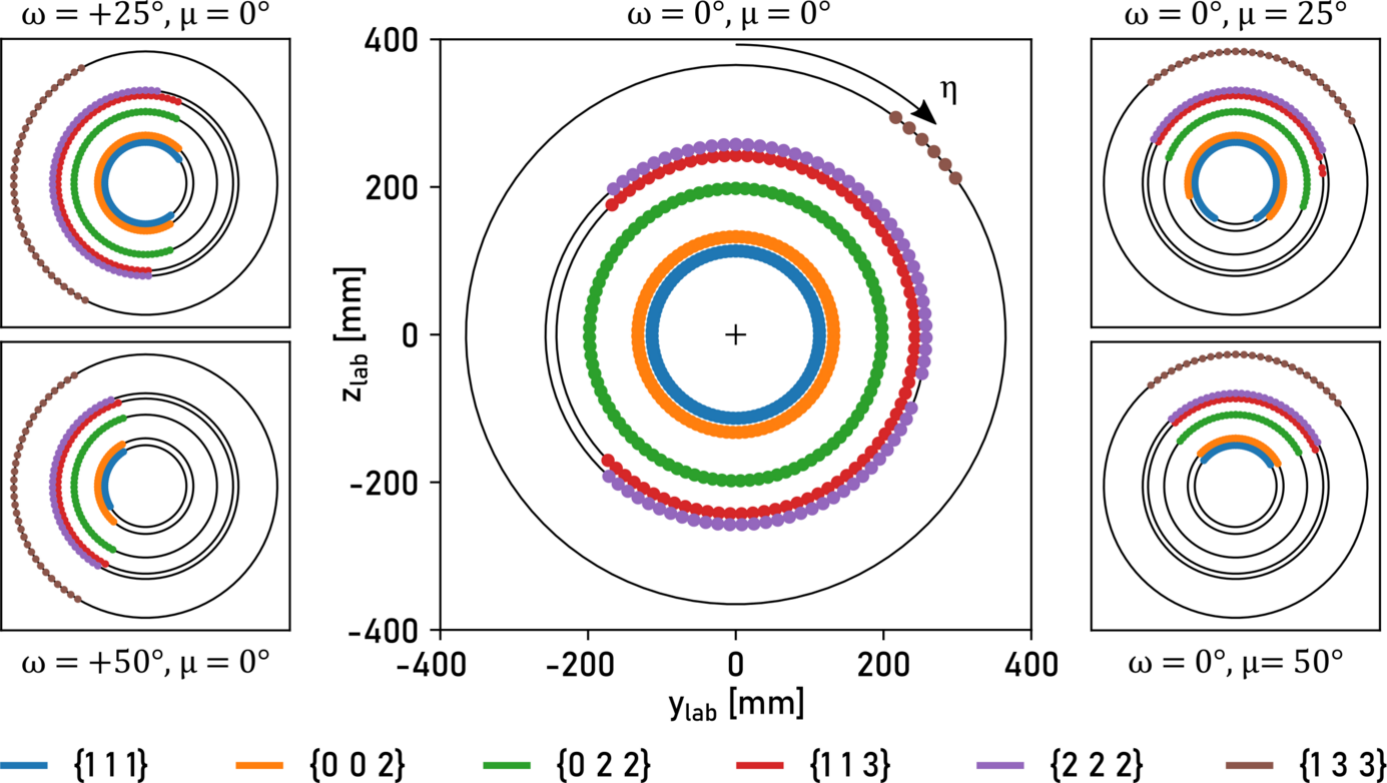
Diffraction ring coverage for several load rig rotations around *y*_lab_ and *z*_lab_ by angles ω and μ [*cf.* Fig. 1 ▸(*a*)]. Plots generated through ray tracing diffraction simulation of face-centred cubic lattice with *a* = 405 pm (aluminium) at an X-ray energy of 17 keV. Dots indicate that the beam is not obscured by the load frame. The detector position is 350 mm downstream parallel to *x*_lab_.

Diffraction techniques such as DFXM require rotating the sample to fulfil the Laue condition of crystallites. The rotation is generally conducted through a stack of goniometers or a hexapod. Inherently, the load frame constrains the solid angle for the incident and diffracted beam, and thus the useful angular range of these rotations as components of the frame may obstruct the beam path. A sketch of the diffraction geometry is shown in Fig. 1 ▸(*a*). To assess the angular range relevant for DFXM, a ray-tracing simulation is performed for aluminium diffraction (*a* = 405 pm) at 17 keV, an energy typically used with beryllium compound refractive lenses serving as the objective. The simulation probes diffracted rays of the first seven lattice-plane families (*i.e.* {111}, {200}, {220}, {311}, {222}, {331} and {333}) in η = 3.6° steps and determines their intersections with the load frame. The size of the simulated incoming beam is 1 mm × 1 mm. At the goniometer’s nominal angular position of (ω = 0°, η = 0°), the first three diffracting lattice plane families ({111}, {200}, {220}) reach the detector for any η, while the {311}, {222} plane families are partially shadowed, as shown in the central plot in Fig. 3 ▸. The higher angle plane families ({331}, and {333}) are blocked entirely. Rotation of the load frame around *z*_lab_ by angle ω [*cf.* Fig. 1 ▸(*a*)] results in increased blockage of the path of the diffracted beam. Diffraction patterns for a series of rotated configurations around *z*_lab_ and *y*_lab_ by angles ω and μ are presented in the same figure. The diffractograms indicate limitations to both rotation operations, as the diffraction signals from the {111} and the {002} families are already partially shadowed by the load frame’s crossheads for rotations of ω = 25° and μ = 25°. The covered η range further decreases as the load frame is rotated to positions ω = 50° and μ = 50°. The angular range is comparable with that of other available setups for diffraction high-load *in situ* studies, such as diamond anvil stages (Kantor *et al.*, 2012 ▸). However, designs specialized for tomographic methods such as 3DXRD often employ transparent materials or rotate the sample inside the setup to avoid mechanical components from the beam path and allow for a full 360° range in ω (Gueninchault *et al.*, 2016 ▸; Shade *et al.*, 2015 ▸).

## 3. Mechanical stability

A central modality of X-ray diffraction techniques is the ability to measure elastic strains by evaluating changes in the *d*-spacing. When measuring elastic strains within the sample (*i.e.* inter- and intra-granular strains), it is important that the setup maintains the sample’s elastic strain, which is usually orders of magnitude smaller than the irreversible plastic deformation. For pure aluminium with a Young’s modulus of *E* ≃ 70 GPa and a flow stress of approximately 10 to 50 MPa, a relaxation of ɛ = 0.015% to ɛ = 0.071% would already result in complete unloading.

**Figure 4:**
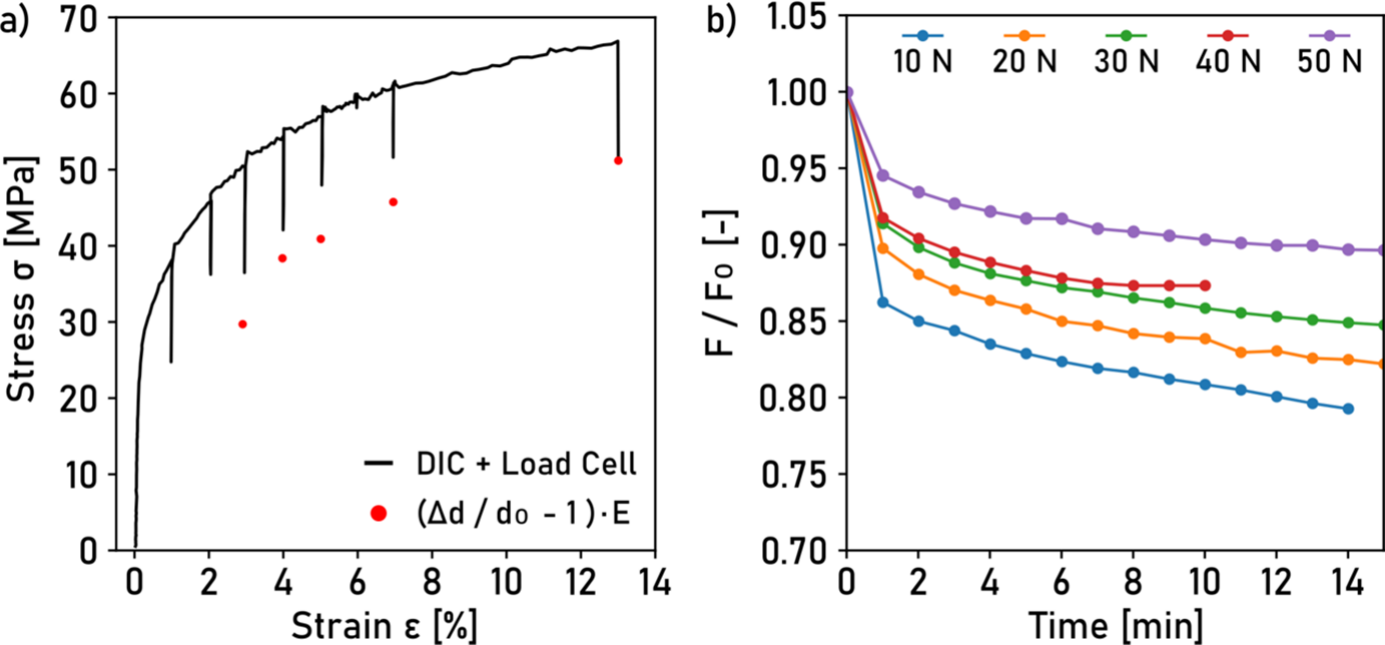
(*a*) Stress–strain curve of the aluminium 1050 sample (DIC and load cell, line) overlaid with elastic strain (Δ*d*/*d*) from 111 *d*-spacings (dots). Load drops stem from pauses for DFXM scans, after which the elastic strain was measured. (*b*) Relative force drop *F*/*F*_0_ as a function of time for several load levels.

The mechanical stability of the setup is assessed through X-ray diffraction. The stress–strain curve shown in Fig. 4 ▸(*a*) was generated with the in-built DIC on an Al1050 tensile sample. The curve is recorded by incrementally straining the sample to ɛ = 15% in step sizes of 0.05% < Δɛ < 0.2%. The loading sequence was interrupted multiple times for 1 to 2 h for DFXM measurements, resulting in significant relaxation, reducing the applied force by approximately 15% measured by the integrated load cell. This relaxation was additionally monitored by tracking changes in the Bragg angle of a single grain’s [111] reflection with an area detector placed 3500 mm from the sample after the DFXM measurement had finished. By comparing the peak positions on the detector with those of the unloaded reference state, the corresponding elastic strains and subsequently the stresses are determined using the Young’s modulus of aluminium along the [111] direction, *E*^111^ = 72.8 GPa (Zhang *et al.*, 2020 ▸). The forces calculated from the measured *d*-spacing indicate a relative relaxation similar to that observed in the load cell readout, suggesting that the recorded reduction in reaction force is directly linked to elastic unloading of the sample.

In a separate test, an identical sample was preloaded to 10, 20, 30, 40 and 50 N, and the drop in reaction force over time was measured by the load cell. The results presented in Fig. 4 ▸(*b*) indicate a relaxation of up to 20% over 15 min. The relative relaxation is decreasing with increasing load levels, which is in agreement with the stress–strain curve shown in Fig. 4 ▸(*a*), suggesting an asymptotic behaviour over time. More than 50% of the relaxation takes place within the first minute after any load increment. This implies that the sample is in a steady state during subsequent experiments. It is noteworthy that deliberate partial unloading is sometimes employed to ensure sample stability (Lienert *et al.*, 2009 ▸).

**Figure 5:**
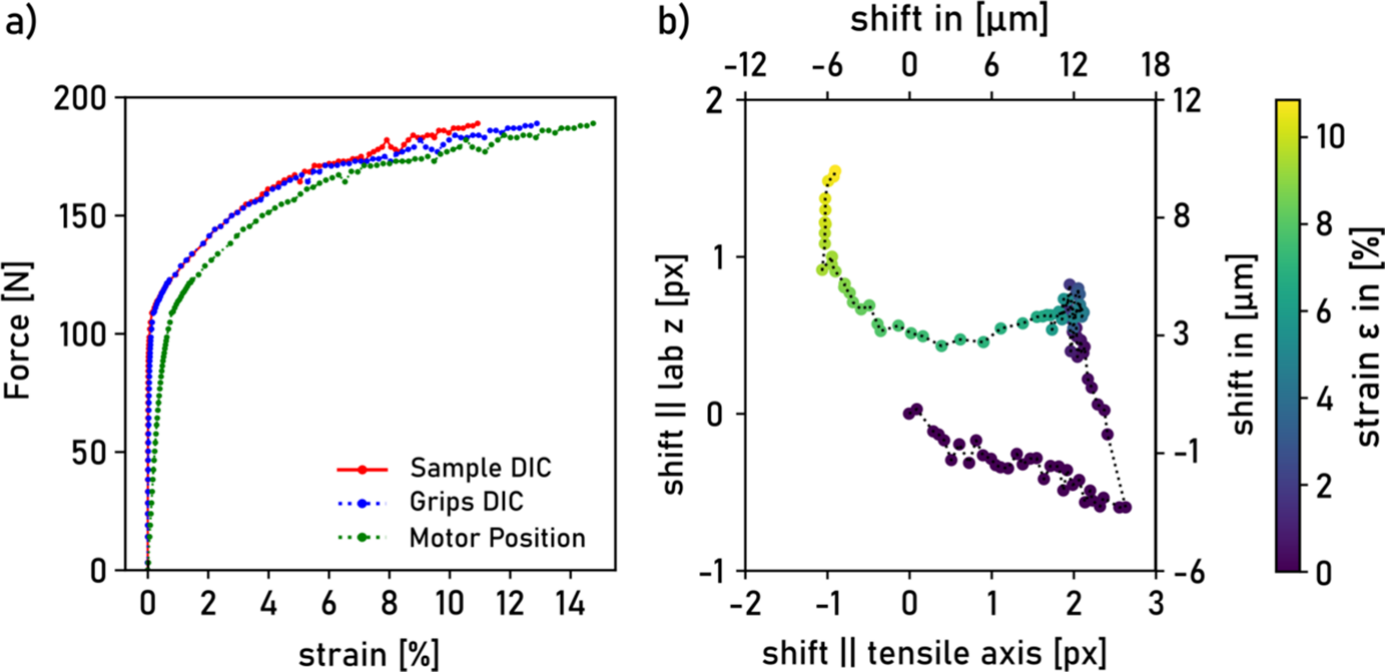
(*a*) Strain–force curve of DC04 steel sample. The force curve is plotted based on the strain calculated from the motor positions and DIC evaluations on the sample grip positions and the gauge area of the specimen. DIC marker positions in Fig. B.4 of the supporting information. (*b*) Shift of the sample centre in the view plane normal to the X-ray beam. The markers are coloured according to the DIC strain value of the load sequence with ɛ ranging from 0% to 11%.

To assess the compliance of the load frame, a load curve with a stiffer steel sample grade DC04 is collected. During the experiment, the sample is strained to 15%, where significant deformation in the dovetails-shaped ends starts to cause load drops. The recorded strain–force curve is shown in Fig. 5 ▸ for the strain based on the motor positions, the sample grip positions and the sample strain. Good agreement between the curves calculated based on the grip positions (blue) and sample elongation (red) indicates that plastic deformation of the sample ends is negligible up to 6%, from whereon both curves begin to diverge. By comparing the lateral offsets of the grip positions (blue) and the motor positions (green), the load frame’s compliance can be estimated. The curves’ relative difference in strain of 12% at the final load step indicates compression of the frame. A plot of the strain offset between the two curves and the force is shown in Fig. B.5 of the supporting information.

The motion of the sample’s central region during step-wise elongation to 11% is shown in Fig. 5 ▸(*b*). This displacement is used to quantify drift of the region of interest under load, assuming no manual correction of the load frame or beam position. The shown curve was acquired using an optical light microscope viewing along the beam axis *x*_lab_ to capture sample movements perpendicular to the beam. The effective pixel size is 6 µm. Although the sample ends undergo a total displacement of more than 500 µm over the loading sequence, the specimen’s centre remains confined to a 24 µm window along the tensile axis. The shift perpendicular to the loading axis, along *z*_lab_, is approximately half at 15 µm. Note that, in this particular case, two changes in shift direction are observed, which are caused by initial settling of the sample in the elastic regime, and plastic deformation in the sample grips at ∼6%. A separate measurement (*cf.* Fig. B.6 of the supporting information) on an Al1050 sample using the DIC camera indicates shifts of similar magnitude along the beam axis of 23 µm. While these shifts exceed the typical DFXM beam height of approximately 500 nm, they remain well below the median grain radius of the material (35 µm), which is a grain size typical for polycrystalline materials, thereby reducing the risk of translating the grain out of the illuminated volume. Possible measures to further reduce drift of the sample’s centre include local slimming of the gauge area and the usage of different mounting geometries, such as pinholes. Furthermore, the stiffness of the setup can be increased either by replacing the trapezoidal nuts in the crossheads with stiffer materials or by using ball-screw assemblies.

## 4. Combination with grain mapping techniques

To put localized intragranular DFXM measurements into the context of grain neighbourhoods, methods to find a specific grain in a pre-indexed grain map from laboratory diffraction contrast tomography (LabDCT) or 3DXRD have been developed (Shukla *et al.*, 2025 ▸). These methods can also be used to assess the accessibility of certain grains and lattice planes, considering the angular coverage of the load frame (*cf*. Fig. 3 ▸).

**Figure 6:**
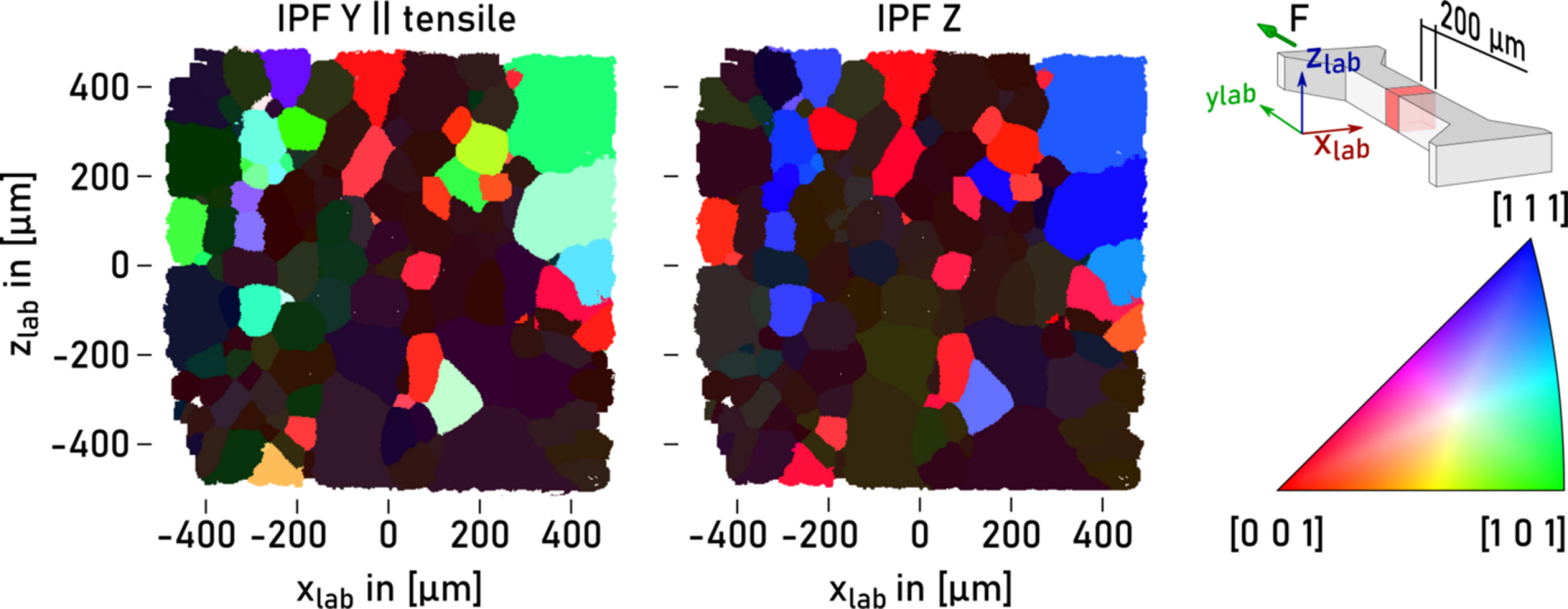
Central layer of the DCT volume coloured by grain orientation parallel to the tensile axis (*y*_lab_) and (*z*_lab_). The latter orientation component is coplanar to the diffraction vector **q** at η = 0°. Grains where no lattice plane is reachable by the DFXM setup are shaded dark.

Such a filtered LabDCT grain map is shown in Fig. 6 ▸ for a sample identical to the one used in the experiment presented in Fig. 4 ▸(*a*). The grain map has been generated without the load frame from a region with a height of 397 µm over the sample’s entire cross section of ∼1 mm^2^. Filtering the map based on the angular coverage of the load frame and the geometry and motor bounds of the DFXM setup at ID03-HXM at the ESRF resulted in 223 of the 724 grains having accessible reflections at an X-ray energy of 17 keV. At that energy, the detector’s range allows imaging the {111} and {200} families of lattice planes. Using objective lens materials suited for higher energies, such as diamond, allows increasing the X-ray energy and accessing additional reflections. For filtering, only lattice planes of grains were considered that result in a diffraction vector coplanar with the *x*–*z* plane in the laboratory reference frame (*i.e.* η = 0°) after rotation within the motor bounds. The rotation motor bounds used for filtering are μ ∈ [−4°; 22°], ω ∈ [−25°; 25°] and χ ∈ [−4°; 4°]. Allowing for an oblique diffraction geometry with η ≠ 0° further increases the subset of reachable reflections.

## 5. Conclusions

In this work, we have presented a remote miniature load frame for X-ray diffraction and imaging applications. The load frame has been assessed for mechanical stability and suitability for various synchrotron X-ray-based techniques. Features of the load frame include:

• Mechanical loads of up to 500 N.

• Partial coverage of the innermost diffraction rings for rotations around *z*_lab_ of ±50° and *y*_lab_ of ±50°.

• Symmetric loading resulting in sample centre shifts <32 µm at ɛ = 15%.

• Force relaxation <20%.

• Integrated digital image correlation and remote controllability.

In outlook, improvements in the angular acceptance will increase compatibility with other tomography techniques, such as computed tomography or diffraction methods, such as 3DXRD or DCT, which require larger angular ranges for ω-rotations.

## Supplementary Material

Supporting Tables A.1 and A.2, and Figures B.1 to B.6. DOI: 10.1107/S1600577526005266/lin5002sup1.pdf

## Data Availability

Software and experimental data will be made available upon request.
