# Supplementary material for: D-stroi – a uniaxial load frame for X-ray diffraction and imaging
Source: J Synchrotron Radiat. 2026 Jun 18;33(Pt 4):1152–8. doi: 10.1107/S1600577526005266 (PMC13344600; doi:10.1107/S1600577526005266)
Supplement: Supplementary file 1 [file s-33-01152-sup1.pdf]

JOURNAL OF SYNCHROTRON RADIATION

Volume 33 (2026)

Supporting information for article:

D-stroi – a uniaxial load frame for X-ray diffraction and imaging

Felix Tristan Frankus, Adam André William Cretton, Carsten Detlefs, Flemming Bjerg Grumsen, Antonella Gayoso Padula and Grethe Winther

Appendix A Components

Description	Manufacturer	Model Number	Quantity
Step Motor	Faulhaber	AM2224R3050030	2
Planetary Gearbox	Faulhaber	Series 26/1R 592:1	2
Lead Screw	IGUS	dryspin PTGSG-08X1.5-01-R/L-1000-ES	2
Lead Screw Nut LH 1	IGUS	JFLM-202020TR8x1.5	1
Lead Screw Nut RH 1	IGUS	JFRM-202020TR8x1.5	1
Lead Screw Nut LH 2	IGUS	JFLM-2020TR8x1.5	1
Lead Screw Nut RH 2	IGUS	JFRM-2020TR8x1.5	1
Ball Bering	-	W 619/5-2Z	4
Guide Shaft	IGUS	drylin R AWMP-10	2
Force Sensor	Althen	ALF259UFR0H0	1
DIC Camera	Dine-Lite	AM8117MZT	1

Table A.1: Bill of Materials – Load Frame

Description	Manufacturer	Model Number	Quantity
Control Microcontroller	Arduino	Uno Rev. 3	1
Motor Controller	STMicroelectronics	X-Nucleo-IHM02A1	1
Load Cell Driver	SparkFun	Qwiic Scale	1
Display	-	HD44780	1
Push-button	-	-	2
Rotary Encoder	-	-	1

Table A.2: Bill of Materials – Motor Control Unit (MCU)

Appendix B Supplementary Figures

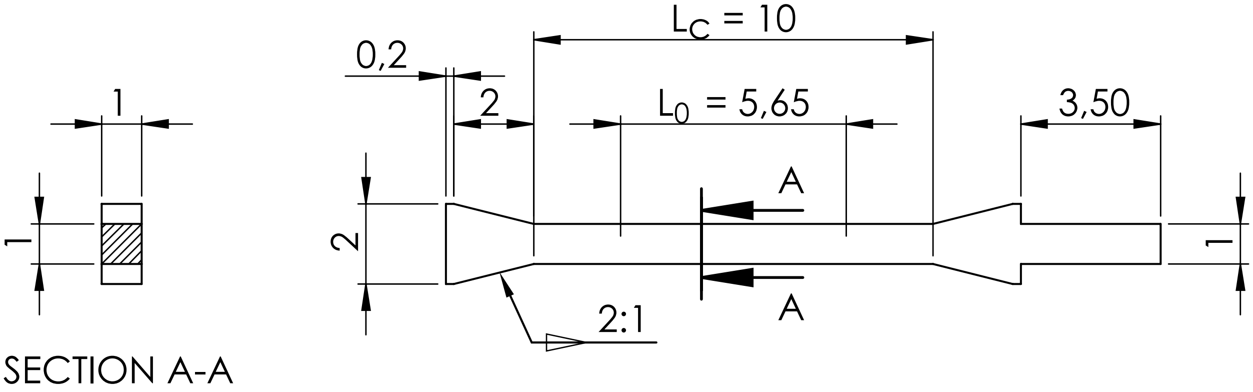

Figure B.1: Technical Drawing of Tensile Specimen.

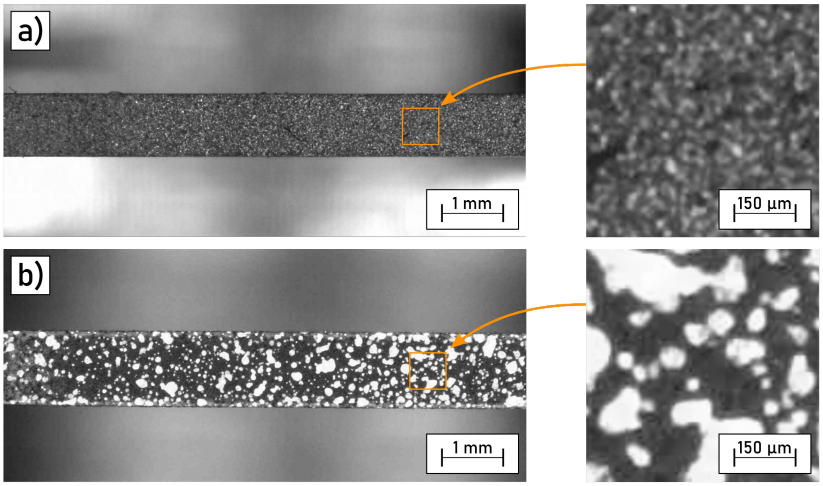

Figure B.2: Camera images used for DIC of sample surface (wire-cut EDM) and random dot pattern of white spray paint applied on a layer of black colour.

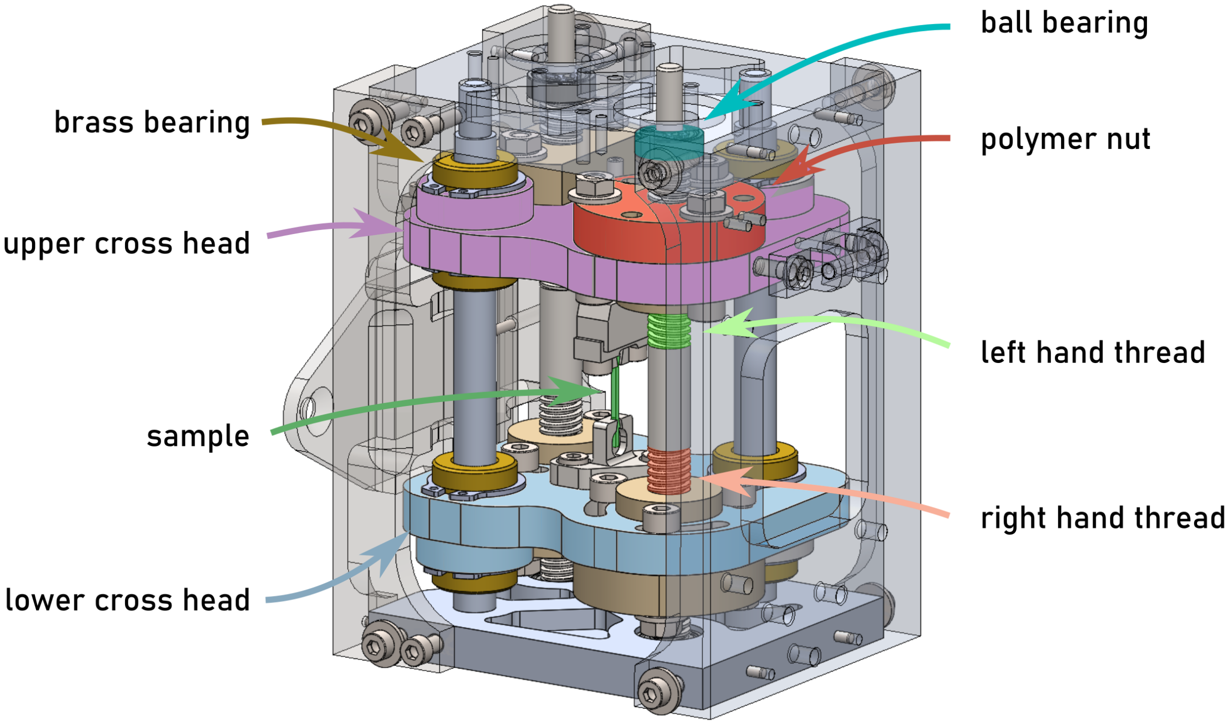

Figure B.3: Rendering of CAD model with highlighted mechanical components.

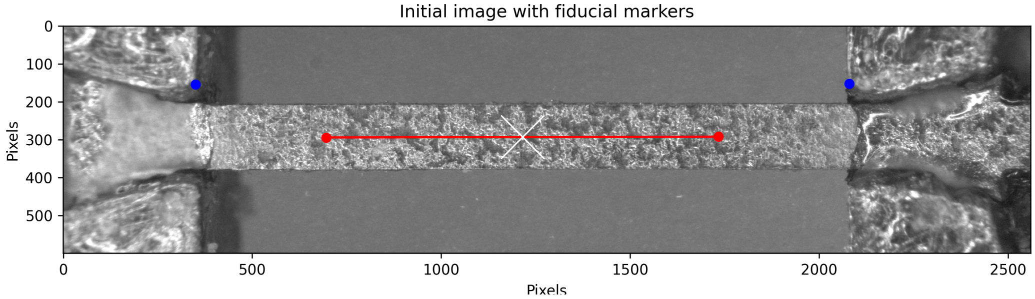

Figure B.4: Initial marker positions for DIC determination of the sample strain (red) and grip positions (blue) for the DC04 sample.

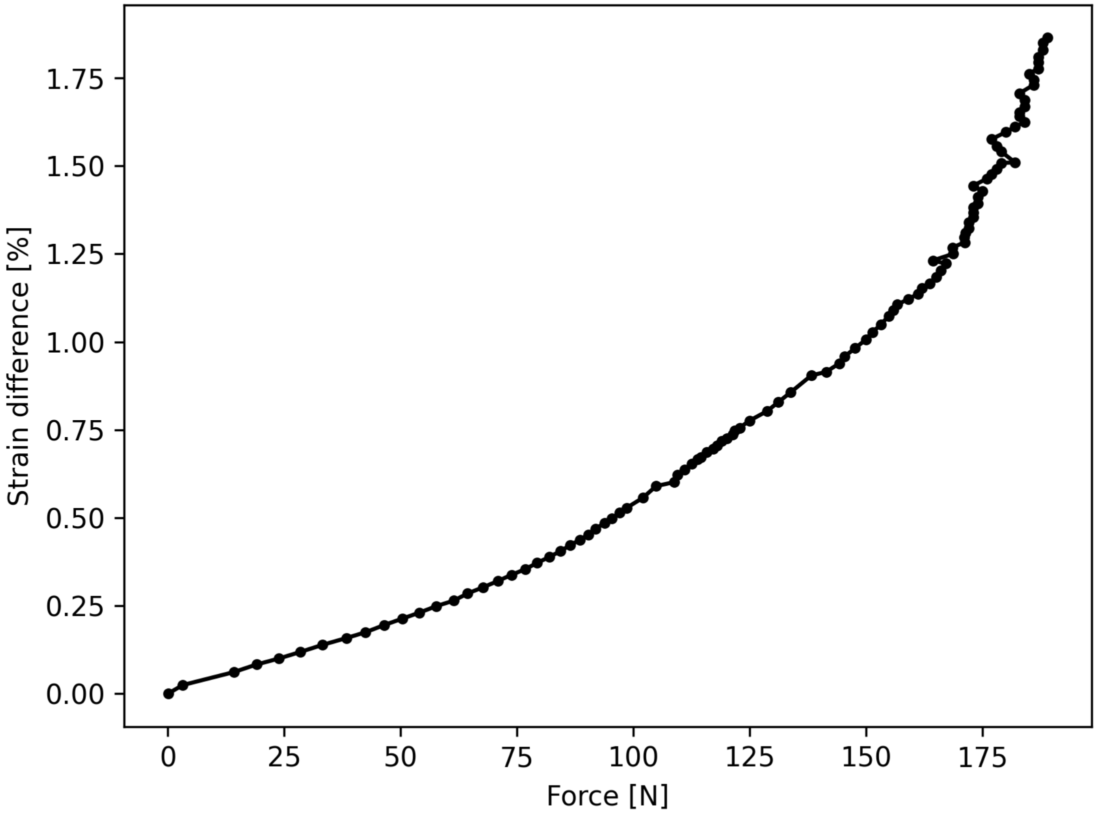

Figure B.5: Difference in strain calculated from motor positions and grip positions as a function of applied load for the tensile test conducted on the DC04 sample. (c.f. Figure 5 in the article)

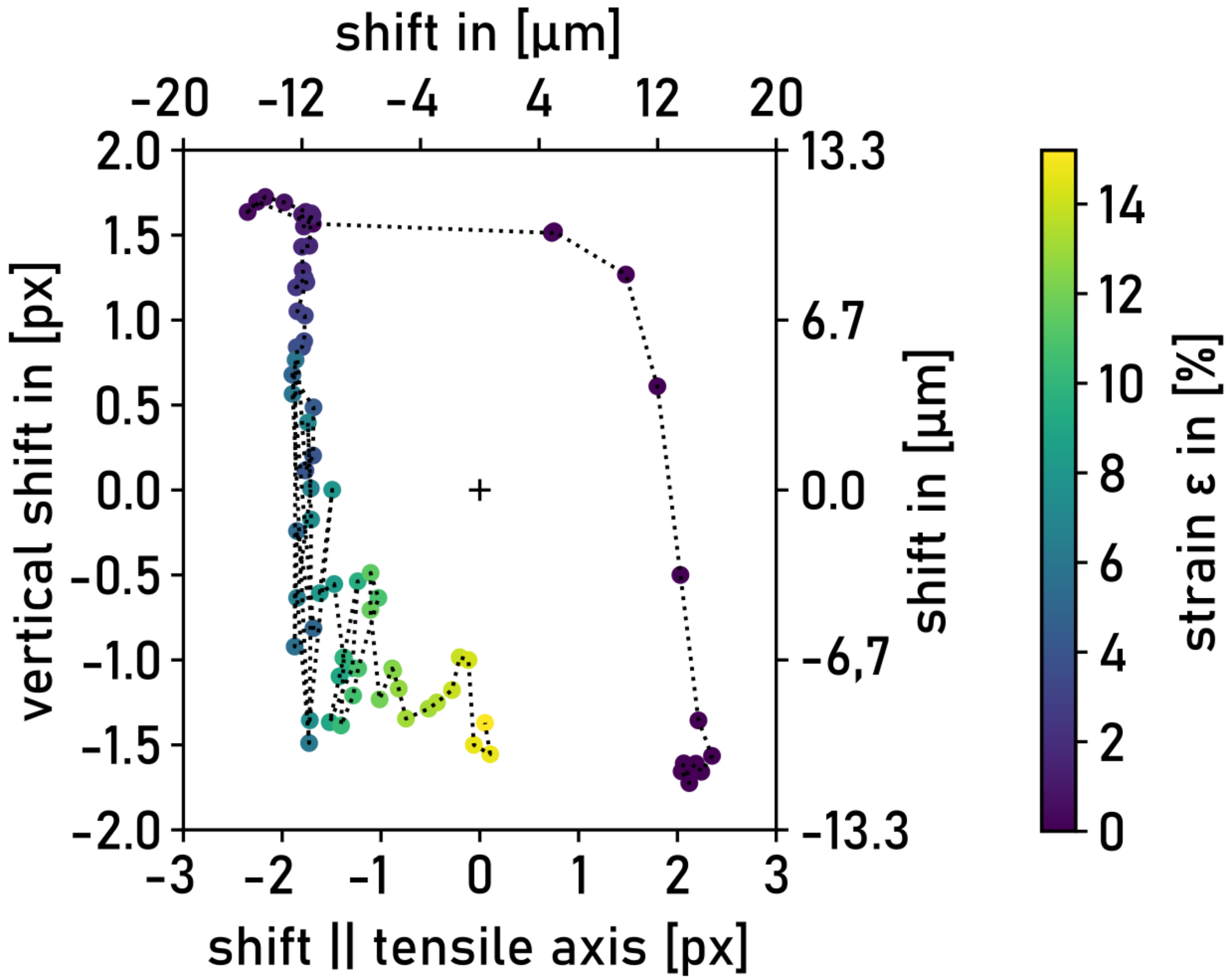

Figure B.6: Drift of the sample centre position of stress-strain curve collected on Aluminium 1050 (c.f. Fig 4a in article) along beam and tensile directions. Markers are colour-coded according to the DIC strain value of the load sequence.
